# Bidirectional Phosphorylation Changes in Opsins Associated With Early Myopia and Hyperopia Signal Regulation by Phosphoproteomics

**DOI:** 10.1167/iovs.66.9.70

**Published:** 2025-07-30

**Authors:** Yang Yang, Ying Hon Sze, Houjiang Zhou, Winky Wing Man Ko, Yuanliang Zhang, Kecheng Li, Qi Zhang, King Kit Li, Trevor C. Charles, Chi-ho To, Qian Zhao, Thomas Chuen Lam

**Affiliations:** 1 Department of Applied Biology and Chemical Technology, The Hong Kong Polytechnic University, Hung Hom, Hong Kong; 2 Centre for Eye and Vision Research (CEVR), Shatin, Hong Kong; 3 Centre for Myopia Research, School of Optometry, The Hong Kong Polytechnic University, Hung Hom, Hong Kong; 4 Zhejiang Hisun Pharmaceutical Co. Ltd., Taizhou, China; 5 Department of Biology, University of Waterloo, Waterloo, Canada; 6 Research Centre for SHARP Vision, The Hong Kong Polytechnic University, Hung Hom, Hong Kong; 7 Research Centre for Chinese Medicine Innovation, The Hong Kong Polytechnic University, Hung Hom, Hong Kong

**Keywords:** myopia, hyperopia, retina, phosphoproteomics, proteomics

## Abstract

**Purpose:**

The study aimed to investigate the role of post-translational modifications (PTMs), specifically phosphorylation, in the pathogenesis of lens-induced myopia (LIM) and lens-induced hyperopia (LIH).

**Methods:**

This study used an untargeted phosphoproteomics approach to identify more than 12,000 phosphorylation sites in chick retinas. The changes in phosphorylation levels were quantified using the tandem mass tag (TMT) technique. Furthermore, targeted mass spectrometry was employed to characterize and validate the phosphorylation changes in visual opsins.

**Results:**

The analysis identified differential phosphorylation at specific sites: S334 in rhodopsin, S328 in violet-sensitive opsin, and S342 in blue-sensitive opsin. Notably, these serine residues were dephosphorylated during the onset of myopia, but they remained phosphorylated under hyperopic conditions. This finding indicates that phosphorylation patterns in opsins are significantly modulated by changes in optical conditions, potentially influencing retinal signaling pathways.

**Conclusions:**

The findings highlight the bidirectional modulation of phosphorylation in opsins as a potential mechanism linking optical factors from induced myopia and hyperopia to the molecular signaling processes that regulate ocular growth and adaptation.

Myopia, a prevalent refractive error, occurs when the eyeball elongates, causing light rays to focus in front of the retina. In contrast, hyperopia arises when the eyeball is too short, resulting in light rays focusing behind the retina. Approximately 30% of the global population is affected by myopia, and hyperopia impacts around 5% to 10% of individuals.^1^^,^^2^ The prevalence of myopia has notably increased, particularly among younger generations, likely due to increases in near work and screen time and reduced outdoor activity.^3^ The World Health Organization estimated that nearly half of the global population will be myopic in 2050.^4^ Myopia is not just limited to blurred vision; it is also a predisposing factor to sight-threatening complications such as glaucoma, cataracts, and retinal detachment.^5^^,^^6^ Researchers widely accept that the retina senses defocus signals and generates biochemical responses that communicate with the choroid and sclera, facilitating tissue remodeling and coordinating axial elongation.^7^^–^^9^ Animal models were employed to mimic human refractive error development to investigate the pathophysiology of myopia.^10^ Among these models, the chick is particularly favored due to its rapid and robust refractive growth response, high visual acuity, wide range of accommodation, and relatively large ocular globes.^10^^–^^12^

Recent advancements in myopia proteomics have led to the proposal of novel ocular proteins and the identification of new biological pathways.^13^ However, how optical signals are decoded at the retina to modulate local ocular changes remains a central unsolved question in myopia research. Post-translational modifications (PTMs), encompassing covalent modifications of proteins during and after biosynthesis, enable cells to respond swiftly to internal and external stimuli.^14^ Among over 400 types of PTMs, phosphorylation is one of the most ubiquitous and plays a crucial role in the ocular system by regulating key cellular processes essential for vision.^15^^,^^16^ Phosphorylation and dephosphorylation of G protein–coupled receptors (GPCRs) are critical regulatory mechanisms across various tissues. For example, phosphorylation modulates signal transduction pathways in photoreceptor cells, affecting GPCR proteins such as rhodopsin (RHO) to enhance phototransduction sensitivity.^17^^,^^18^ Additionally, phosphorylation facilitates intercellular communication and governs the proliferation and differentiation of ocular tissues.^19^^,^^20^ Dysregulation of phosphorylation has been implicated in various ocular diseases, such as diabetic retinopathy and glaucoma, underscoring its significance in maintaining ocular health.^21^ Limited reports investigating phosphorylation events, specifically in myopia and hyperopia, represent a significant knowledge blind zone in our understanding of the molecular mechanisms underlying these refractive errors in the central dogma of photoreceptors and opsins.

This study employed a phosphoproteomics approach to investigate the molecular mechanisms driving myopia and hyperopia progression. By comparative analysis of two distinct ocular growth phenomena, our analysis revealed alterations in phosphorylation levels among three photosensitive opsins: RHO, violet-sensitive opsin (OPSV), and blue-sensitive opsin (OPSB). Notably, we observed bidirectional modulation of phosphorylation between myopia and hyperopia models, highlighting the proposed roles these opsins play in refractive error development. Using targeted mass spectrometry techniques, we validated homologous phosphorylation sites located near the C-terminus, specifically differential phosphorylation and dephosphorylation at S334 of RHO, S328 of OPSV, and S342 of OPSB. Our findings provide new perspectives on optical signal transduction and biochemical signal processing within the retina and may suggest novel therapeutic interventions to address refractive errors.

## Methods

### Animals

White Leghorn chick specific pathogen-free eggs (*Gallus* *gallus*) were obtained from Jinan Poultry Co. Ltd. (Jinan, China). Eggs were housed in an egg incubator (ELYE-3; Onelye, Wuxi, China) for 21 days with an average temperature of 36.6°C and humidity of 68 g/kg in the centralized animal facilities at Hong Kong Polytechnic University. Eggs were moved to a hatcher (EH-96H; Onelye) for 1 week under identical temperature and humidity conditions. Newborn chicks at 3 days of age were housed in stainless steel brooders under a 12-hour light/12-hour dark cycle with an average luminance of 500 lux at the center of the cage inside the breeding room, with a computer-controlled humidity of 41% at room temperature and free access to food and water. Researchers were licensed by the Department of Health, Hong Kong Special Administrative Region. All procedures performed in this study received ethics approval from the Animal Subjects Ethics Sub-Committee and The Hong Kong Polytechnic University and complied with the ARVO Statement for the Use of Animals for Ophthalmology and Vision Research.

### Experimentally Induced Myopia or Hyperopia in Chicks

White Leghorn chicks at the age of 7 days were subjected to randomized eye, unilateral lens-induced myopia (LIM) using a dispersive lens (–10 diopter [D]) or lens-induced hyperopia (LIH) using a convex lens (+10 D), both made from poly(methyl methacrylate) (PMMA) material. Each lens was attached to the chick using a Velcro ring glued to feathers in the area around the eye using epoxy resin a day ahead. Spectacles were cleaned daily to ensure proper vision. Ocular biometric measurements were taken on day 7 (D0) and after 3 days of optical treatments (days of treatment) on day 10 (D3). First, we applied a tandem mass tag (TMT)-based quantitative phosphoproteomics strategy to discover the differentially expressed phosphopeptides in two animal models under LIM and LIH separately. Chicks (*n* = 5) were subjected to randomized unilateral treatment with LIM, and the contralateral control eye (CTL) maintained uninterrupted vision. Additionally, chicks (*n* = 5) received randomized unilateral treatment with LIH with their CTL eyes as controls. Second, we employed a targeted mass spectrometry (MS) approach to validate the differentially expressed phosphopeptides in the phosphoproteomics results using two independent groups of chicks with LIM (*n* = 7) and LIH (*n* = 6).

### Ocular Biometric Measurements in Chicks

Refractive error was measured by an experienced optometrist using a streak retinoscope (Heine Beta 200; HEINE Optotechnik, Gilching, Germany) with a trail lens bar (±16 D in 0.5-D steps) in a dim-light environment with 20-cm distance from the hand-held retinoscope. Equivalent sphere measurements defined the refractive error as spherical power ± 0.5 cylindrical power. Vertical and equatorial axes were measured twice; each measurement was represented as the mean value in diopters ± SD and repeated in triplicate. Ocular dimensions were measured with an A-scan ultrasound system (5073PR; Olympus, Tokyo, Japan) coupled with a 30-MHz probe (PZ25-025-R1.00; Panametrics, Billerica, MA, USA) and an adjustable pump system (505u; Watson Marlow, Falmouth, UK). Saline was used as liquid media in the ultrasonic probe for chick eyes, and deionized water was used for system calibration. A custom-made eyelid retractor was used to keep the chick eyes open during the A-scan measurement without anesthesia, which were acquired in triplicate. The axial length (AL) of the eye is comprised of anterior chamber depth (ACD), lens thickness (LT), and vitreous chamber depth (VCD).

### Isolation of Neural Retina in Chick Eyes

Chicks were sacrificed with carbon dioxide overdose. Extraocular muscles and optic nerves were disconnected by small incisions by scissors, and the eyeball was isolated from the orbit. The intact eyeball was rinsed with ice-cold PBS to remove excessive muscles and blood. The eyeball was hemisected equatorially using a stainless-steel razor blade, dissecting the eyeball into two hemispheres. The crystalline lens was pulled out with forceps. The posterior eyeball was submerged into new, clean, ice-cold PBS and shaken gently using forceps to hold the edge of the choroidal sclera tissue. Pale-yellow, transparent neural retina tissue separated from the posterior eyeball, leaving the retinal pigment epithelium layer with choroidal–sclera tissue. The isolated retinal tissue was snap-frozen in liquid nitrogen and stored in a −80°C freezer.

### Tissue Homogenization and Protein Extraction

Chick retina tissues were harvested and immediately frozen in liquid nitrogen. The tissues were then homogenized in 250 µL 100-mM Tris-HCl, 7-M urea, 1-mM MgCl_2_, 1% benzonase, 1-mM sodium orthovanadate, 1× PhosSTOP phosphatases inhibitor, and 1× EDTA-free protease inhibitor. The tissues were homogenized with an automated homogenizer (Bertin Technologies, Paris, France) by running four cycles of 10 seconds at 5800 rpm, with a 30-second break between cycles. Residual debris removal was performed by centrifugation at 12,000 rpm for 30 minutes at 4°C. The supernatant was collected for reduction and alkylation. Proteins were reduced by 5-mM dithiothreitol for 30 minutes at 37°C with gentle shaking and then incubated with 15-mM iodoacetamide at room temperature for 30 minutes in the dark. In-solution digestion began with mass spectrometry (MS)-grade Lys-C (FUJIFILM Wako, Richmond, VA, USA) at an enzyme/protein ratio of 1:200 (w/w) at 37°C. A secondary digestion was performed by adding sequencing-grade trypsin (Promega, Madison, WI, USA) at an enzyme/protein ratio of 1:50 (w/w) for an additional 12 hours at 37°C. Peptides were acidified and desalted with a Sep-Pak C18 cartridge (Waters, Milford, MA, USA). The samples were dried by a concentrator and stored in a −80°C freezer.

### Phosphorylated Peptide Enrichment and TMT Labeling

Phosphorylated peptide enrichment was performed using TiO_2_ according to the method previously described.^22^^,^^23^ The sample loading buffer was 50% acetonitrile (ACN) and 2-M lactic acid; the washing buffer was 50% ACN and 0.1% TFA; elution buffer 1 was 10% NH_3_·H_2_O; and elution buffer 2 was 5% NH_3_·H_2_O and 50% ACN. The enriched phosphorylated peptides were dried and reconstituted in 50 µL 100-mM triethylammonium bicarbonate (TEAB; pH 8.5). The peptide concentration was determined with a quantitative fluorometric peptide assay kit (Thermo Fisher Scientific, Waltham, MA, USA). The TMT labeling was performed according to the manufacturer with a TMT/peptide ratio of 10:1 (w/w).

### Liquid Chromatography With Tandem Mass Spectrometry for Phosphoproteomics Analyses

Liquid chromatography with tandem mass spectrometry (LC-MS/MS) analyses of retina samples were performed on an Orbitrap Exploris 480 mass spectrometer (Thermo Fisher Scientific) coupled with an ultra-performance liquid chromatography (UPLC) system (UltiMate 3000; Thermo Fisher Scientific). A reversed-phase liquid chromatography (RSLC) analytical column (C18, 75 µm × 250 mm, 2.0 µm, 100 Å; Thermo Fisher Scientific) was employed for liquid chromatographic separation. Mobile phase A is 0.1% formic acid (FA) in water, and mobile phase B is 0.1% FA in 80% ACN. A 180-minute gradient with a 300-nL/min flow rate and an initial 8% mobile phase B was used. Mobile phase B was increased to 32% at 130 minutes and 90% at 158 minutes and was held for 10 minutes. Then, mobile phase B was changed back to 8% at 170 minutes, and this composition was maintained until 180 minutes. Data were collected in data-dependent acquisition mode. The top 10 precursor ions with a charge state of 2+ or higher were fragmented by higher energy collisional dissociation. The precursor ion (MS1) Orbitrap resolution was set at 60,000, and the MS1 automatic gain control (AGC) target was 4 × 105. The product ion (MS2) Orbitrap resolution was set at 30,000, and the MS2 AGC target and maximum injection time were set at 1 × 105 and 54 ms, respectively. TMT data were searched using the reference proteome of *Gallus* *gallus* (chicken) in the UniProt database with entry identifier UP000000539 with the SEQUEST algorithm (Proteome Discoverer 2.4; Thermo Fisher Scientific). Carbamidomethylation of cysteine residues was set as a static modification. TMT tags on lysine residues and the peptide N-terminal, phosphorylation of serine/threonine/tyrosine, and oxidation of methionine residues were set as variable modifications.

### Target Validation With Parallel Reaction Monitoring Strategy

Parallel reaction monitoring (PRM) analyses of retina samples were performed on the Orbitrap Exploris 480 mass spectrometer coupled with the UltiMate 3000 UPLC system. An RSLC C18 analytical column (75 µm × 250 mm, 1.6 µm, 120 Å; IonOpticks, Collingwood, Australia) was employed for liquid chromatographic separation. A 120-minute gradient with a 300-nL/min flow rate and an initial 8% mobile phase B was used. Mobile phase B was increased to 32% at 80 minutes and 90% at 98 minutes and was then held for 10 minutes. Mobile phase B was then set back to 8% at 110 minutes, and this composition was maintained until 120 minutes. The targeted MS1 parameters were as follows: resolution, 120,000; AGC target, 4.0 × 105; and maximum injection time, 100 ms. MS2 scanning was performed at 60,000 resolution, 1 × 105 AGC target, and 1.0 mass-to-charge ratio (m/z) isolation window.

### Bioinformatics Analysis

After the PRM data acquisition, the data were imported into Skyline 24.1 (University of Washington, Seattle, WA, USA) for analysis. For reliable identification and quantification, the values of isotope dot product (idotp) and dot product (dotp) values should exceed 0.70. The top three product ions were summed up to represent the peptide abundance. The idotp measures the similarity between experimental and theoretical isotope distributions, and the dotp assesses the match between experimental and reference fragment ion spectra. Higher idotp scores and higher idotp scores reflect the confidence of identifications in targeted proteomics. The top three product ions were summed up to represent the peptide abundance. The Gene Ontology (GO) analysis was performed by DAVID bioinformatics for functional annotation (https://davidbioinformatics.nih.gov/tools.jsp). Structural predictions were performed on the AlphaFold server by DeepMind.

## Results

### Bidirectional Regulations of Axial Length in Chick Eyes With Spectacle Lenses

**Figure 1.**
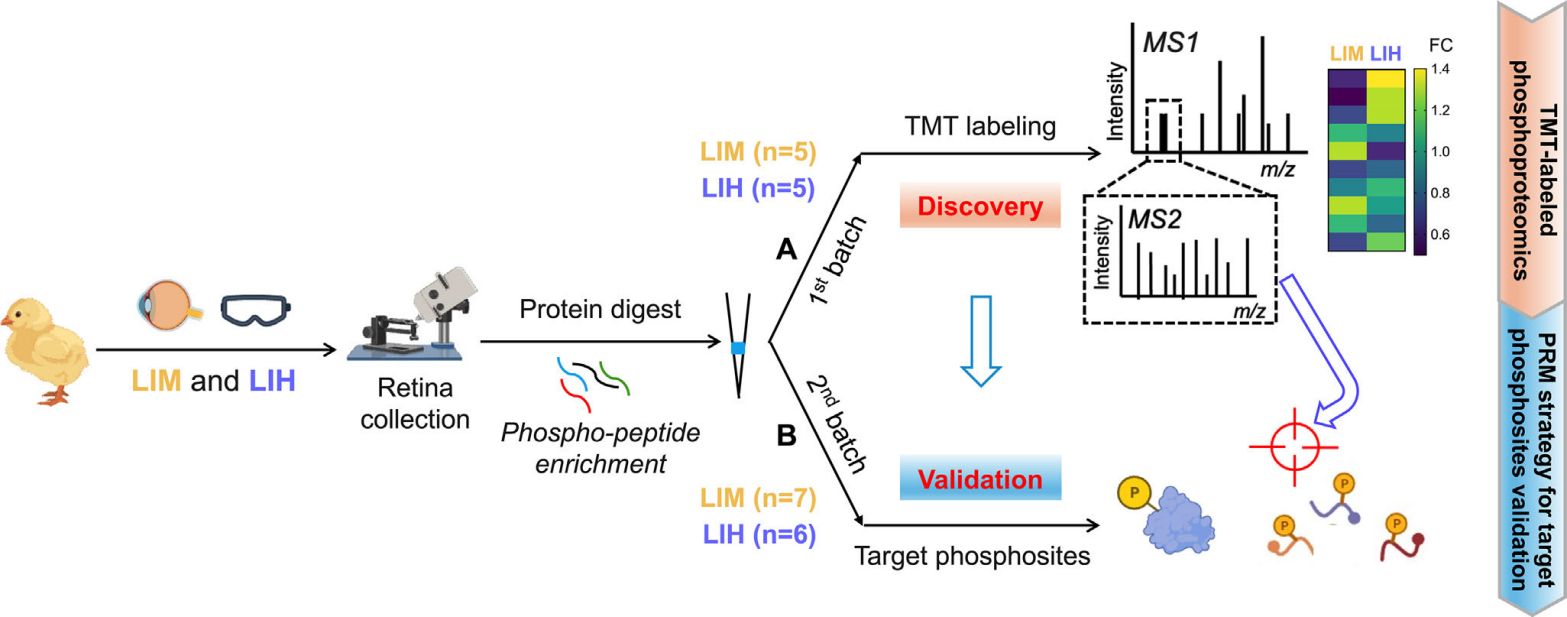
Schematic workflow of the phosphoproteomics study. Collected retina samples from LIM, LIH, and the corresponding control were homogenized and lysed for protein extraction, followed by protein digestion, in-house phosphopeptide enrichment, and LC-MS/MS analysis for phosphorylation site identification. (**A**) The TMT-labeled phosphoproteomics study was performed in chick retinae subjected to LIM (*n* = 5) and LIH (*n* = 5) treatment to identify differentially phosphorylated peptides. (**B**) The PRM strategy utilizing the MS2 mass spectra to validate precise phosphorylation sites in opsins was conducted in chick retinae subjected to LIM (*n* = 7) and LIH (*n* = 6) treatment.

**Figure 2.**
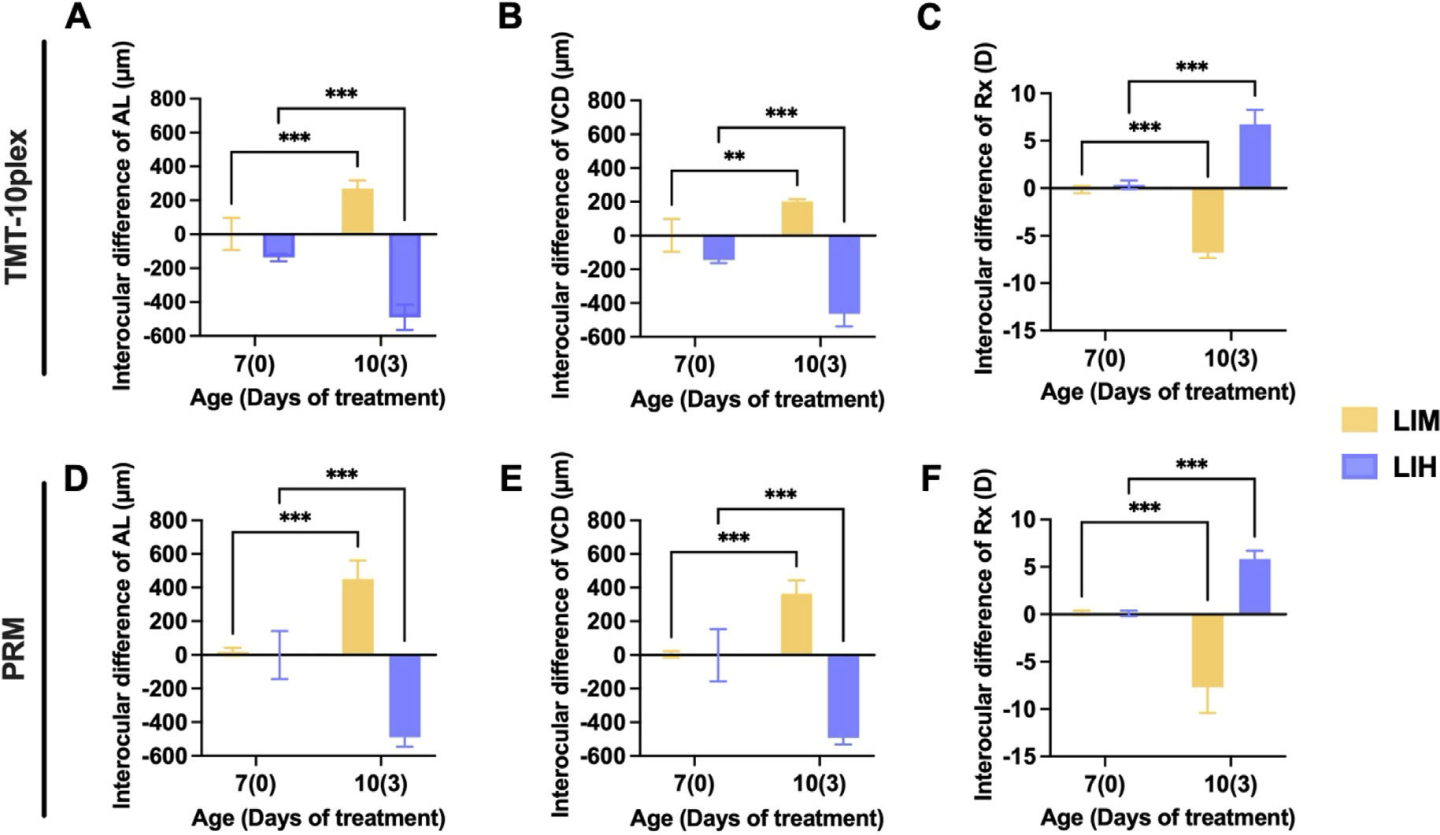
Ocular biometric characteristics of chicks subjected to LIM and LIH treatments. Comparison of interocular differences between chicks at day 7 and day 10 after 3 days of optical treatment. (**A**–**C**) The distinctive features of the LIM (*n* = 5) and LIH (*n* = 5) models are illustrated with interocular differences in AL (*left*), VCD (*middle*), and refractive error (Rx, *right*). (**D**–**F**) Two groups of chick eyes were prepared for the TMT10plex experiment in the LIM group (*n* = 7) and LIH group (*n* = 6). Two independent replications were prepared for the PRM experiment. Statistical analysis on interocular differences was conducted using two-way ANOVA with Bonferroni correction. **P < 0.01, ***P < 0.001.

For the chick samples used in the discovery experiment (Fig. 1A), the ocular biometric results revealed distinct interocular differences between the treated and control eyes induced by the imposition of a –10 D (concave) lens for myopia induction and a +10 D (convex) lens to induce hyperopia experimentally. There were significant interocular differences in AL in the LIM group, with the LIM-treated eye measuring 267.3 ± 38.2 µm longer than the control eye (*P <* 0.001). Conversely, the LIH group exhibited significantly shorter AL, with the LIH-treated eye being 353.6 ± 38.2 µm shorter than the control eye (*P <* 0.001) (Fig. 2A). The VCD was significantly longer in the LIM-treated eye (199.9 ± 36.6 µm; *P <* 0.01), and the LIH-treated eye had a significantly shorter VCD (320.4 ± 36.6 µm; *P <* 0.001) (Fig. 2B). These observed VCD differences accounted for 75% of axial elongation in the LIM model and 90% of axial shortening in the LIH model. Refractive error measurements indicated a significant myopic shift in the LIM-treated eyes (–6.65 ± 0.49 D; *P <* 0.001) and a hyperopic shift in the LIH-treated eyes (+6.4 ± 0.49 D; *P <* 0.001) (Fig. 2C). Additionally, for the chick samples used in the validation experiment (Fig. 1B), the ocular biometric results reaffirmed the interocular differences induced by experimental myopia and hyperopia. Significant interocular differences included the LIM-treated eye being 431.7 ± 45.7 µm longer than the control eye (*P <* 0.001) and the LIH-treated eye measuring 489.2 ± 49.4 µm shorter than the control eye (*P <* 0.001) (Fig. 2D). VCD measurements revealed significant differences, with the VCD in the LIM-treated eye being longer than the control eye (360.5 ± 43.6 µm; *P <* 0.01) and significantly shorter in the LIH-treated eye (490.8 ± 47.1 µm; *P <* 0.001) (Fig. 2E). These VCD differences accounted for 84% of axial elongation in the LIM model and 100% of axial shortening in the LIH model. Refractive error assessments showed a significant myopic shift in the LIM-treated eyes (–7.9 ± 0.76 D; *P <* 0.001) and a hyperopic shift in the LIH-treated eyes (+5.8 ± 0.82 D; *P <* 0.001) (Fig. 2F). Meanwhile, there were no significant differences in body weights between experiment groups and minimal differences in the axial ocular segmentation, including ACD and LT by both optical treatments (Supplementary Fig. S1). In summary, these four independent groups of animal experiments consistently demonstrated reproducible observations of axial elongation, changes in VCD, and refractive shifts in the LIM and LIH models. These ocular biometric measurements provided physiological context for the subsequent phosphoproteomics analyses.

### Quantitative Phosphoproteomics Revealed Phosphorylation Alterations in LIM and LIH

**Figure 3.**
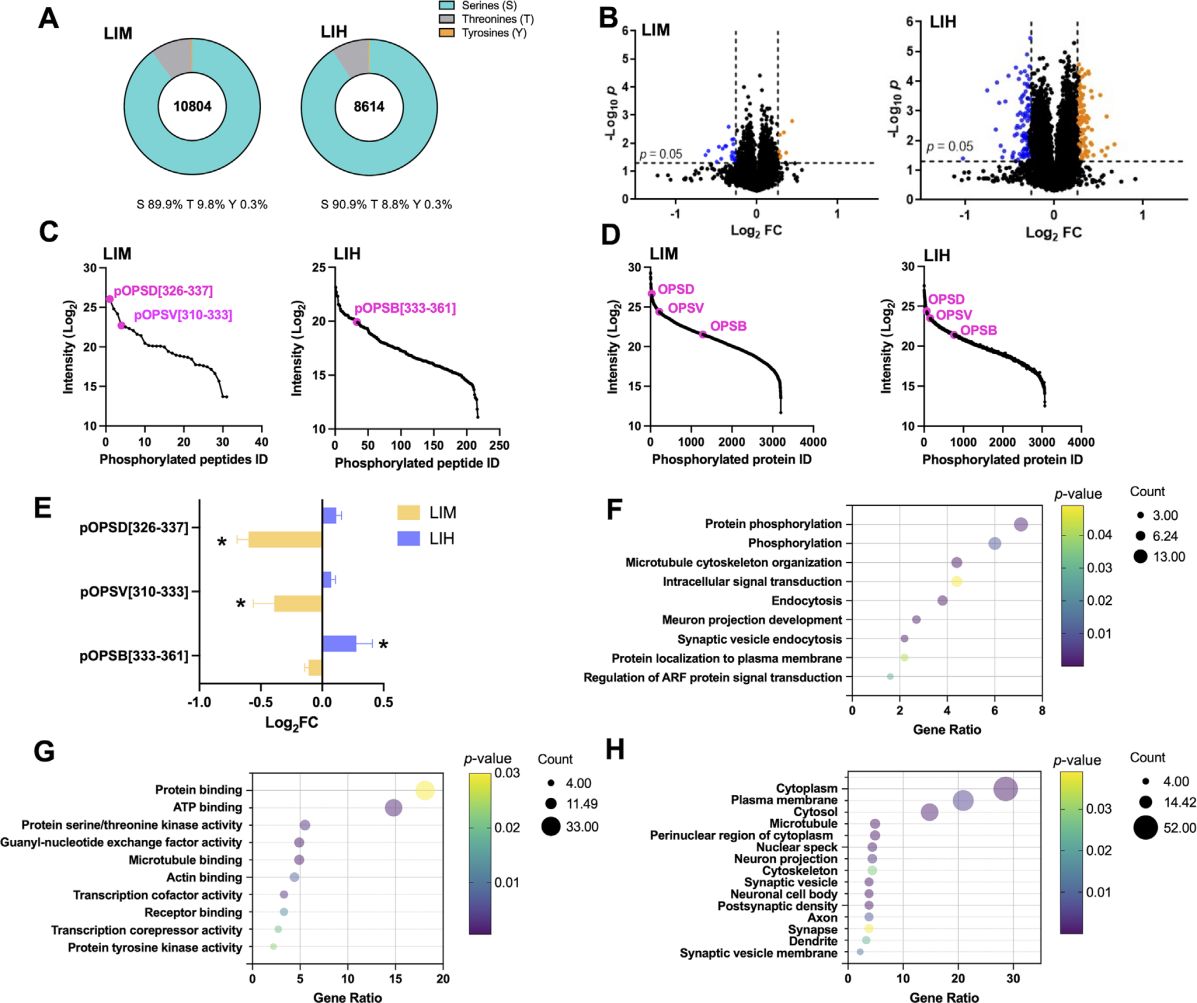
Quantitative phosphoproteomics revealed the phosphorylation changes of opsins in myopia and hyperopia development. (**A**) Identification of phosphorylation sites in LIM and LIH. (**B**) Volcano plots of quantified phosphopeptides identified by MS. Phosphopeptides with fold-changes (FC, LIM/CM, or LIH/CH ratio) >1.2 or < 0.83 (*P <* 0.05) were used. *Orange dots* indicate the upregulated phosphopeptides, and *blue dots* represent the downregulated phosphopeptides with statistical significance. (**C**, **D**) Relative abundance ranks of differentially expressed phosphopeptides (**C**) and phosphoproteins (**D**) in LIM and LIH. (**E**) Phosphoproteomics results revealed the reversed trend of phosphorylation level of three phosphopeptides in LIM and LIH (*P < 0.05). (**F**–**H**) Results of GO analyses of biological processes (**F**), molecular function (**G**), and cellular component categories (**H**) on differentially abundant phosphoproteins from LIM and LIH revealed significant insights into the underlying mechanisms of myopia and hyperopia.

We optimized the ratio of titanium dioxide (TiO_2_) to peptide (w/w) to achieve optimal performance in phosphorylated peptide enrichment from chick retinas with an optimal ratio of 4.0. This ratio yielded the highest number of identified phosphopeptides and the most substantial enrichment percentage (Supplementary Fig. S2). We employed TMT to label the peptides for subsequent phosphoproteomics studies to facilitate multiplexed quantification across samples. Retina samples were collected under LIM or LIH conditions, with the contralateral eye as the control. The phosphoproteomics approach enabled the identification and quantification of 10,804 unique phosphopeptides in LIM samples predominantly phosphorylated on serine (pSer, 89.9%), followed by threonine (pThr, 9.8%) and tyrosine (pTyr, 0.3%). Similarly, in LIH samples, 8614 unique phosphopeptides were identified (90.9% pSer, 8.8% pThr, and 0.3% pTyr) (Fig. 3A). Applying a fold-change (FC) cutoff of 1.2 and a significance level of *P* < 0.05, we found seven upregulated and 19 downregulated phosphopeptides in LIM, whereas, in LIH, we found 105 upregulated and 110 downregulated phosphopeptides (Fig. 3B). Interestingly, when we ranked significantly changed phosphopeptides in LIM by their signal intensities, phosphopeptides from opsins, specifically, the ^326^NPLGDEDTSAGK^337^ of RHO and ^310^ACIMETVCGKPLTDDSDASTSAQR^333^ of OPSV, occupied top positions (Fig. 3C). Similarly, in LIH, the phosphopeptide ^333^SPFGDDEDVSGSSQATQVSSVSSSHVAPA^361^ of OPSB was among the most significantly changed phosphopeptides. Notably, their corresponding proteins ranked in the top 20% of signal intensities over 3000 phosphoproteins quantified in LIM and LIH (Fig. 3D). Interestingly, we observed reversed trends in the phosphorylation levels of these three opsin-derived phosphopeptides between the LIM and LIH models (Fig. 3E). The observed distinct phosphorylation patterns between LIM and LIH models suggest a possible pivotal role of opsin phosphorylation in refractive error development.

To understand further the biological implications of the altered phosphoproteome, we performed a GO analysis on proteins linked to significantly changed phosphopeptides in LIM and LIH (Figs. 3F–3H). The analysis indicated the prominence of phosphorylation as a key biological process critical for regulating several signaling pathways vital for retinal function.^24^ Notably, we observed key phosphorylation-dependent pathways such as the phototransduction cascade, which is known to modulate refractive development where RHO and opsins play critical roles. Our phosphoproteomics data also highlighted prominent opsin phosphorylation under LIM and LIH. These findings suggest that phosphorylation may serve as a central regulatory mechanism of retinal function, potentially through light-dependent activation of protein kinase cascades and regulating opsins that influence both structural and functional adaptations in the retina. Additionally, protein binding and adenosine triphosphate binding emerged as the top two molecular functions, emphasizing the significance of these interactions in facilitating essential biochemical processes such as energy transfer and molecular signaling. These findings suggest that the intricate interplay between phosphorylation and binding activities is fundamental to the functional dynamics of retinal proteins, potentially influencing visual signal transduction and adaptation mechanisms in visually guided eye growth.

### Validation of the Opsin Phosphorylation Sites With Targeted MS

**Figure 4.**
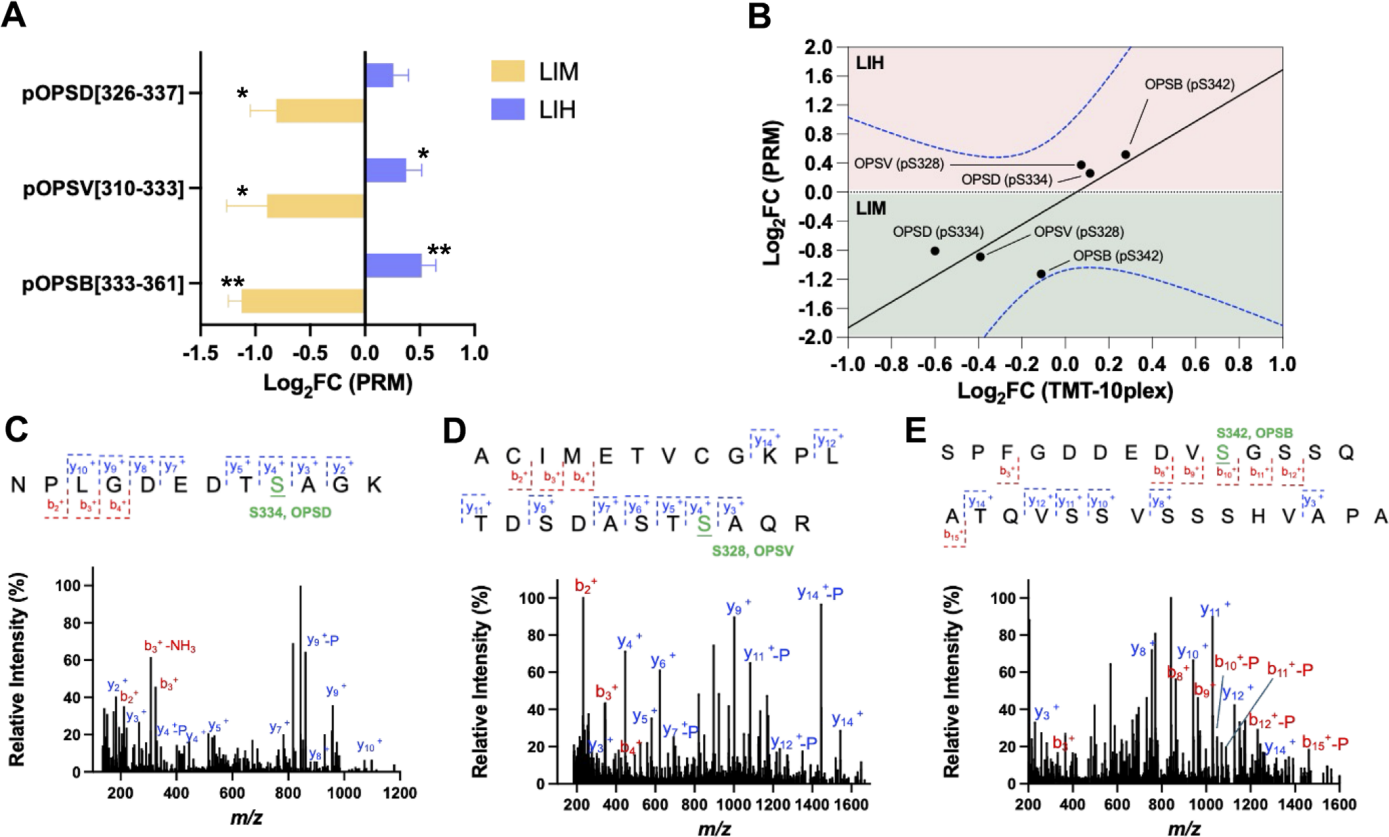
Validation of phosphorylation sites of opsins with targeted mass spectrometry. (**A**) Targeted MS verified the reversed trend of the phosphorylation level of three phosphopeptides in LIM and LIH. *P < 0.05, **P < 0.01. Log_2_FC represents the ratio of LIM or LIH over controls. Chicks were used for LIM (*n* = 7) and LIH (*n* = 6). (**B**) Correlation between FCs of opsins in PRM and TMT datasets with 99% confidence intervals (*blue dotted lines*). Representative MS/MS spectra evidence identified the phosphorylation sites of opsins. (**C**) Phosphopeptide NPLGDEDTSAGK of the rhodopsin. (**D**) Phosphopeptide ACIMETVCGKPLTDDSDASTSAQR of the violet-sensitive opsin. (**E**) Phosphopeptide SPFGDDEDVSGSSQATQVSSVSSSHVAPA of the blue-sensitive opsin.

We investigated the phosphorylation sites of opsins using an alternative MS method, targeted MS, or PRM. The MS intensities of the opsin phosphopeptides showed downregulation during LIM and upregulation in LIH, revealing dephosphorylation during myopia onset in contrast to phosphorylation in hyperopic conditions (Fig. 4A). Notably, among the three phosphopeptides examined, the change in the phosphopeptide of OPSB exhibited the most prominent shifts. Moreover, we observed a positive correlation between the TMT and the targeted MS results in both LIM and LIH conditions (Fig. 4B), reaffirming our observations of the bidirectional modulation of phosphorylation in opsins during myopia and hyperopia. We identified phosphorylation sites by tracking two key features in the mass spectra. First, the neutral loss of phosphoric acid, H_3_PO_4_, with a m/z of 97.977 Da, often occurs during collisional fragmentation and is considered a phosphorylation signature. Second, the characteristic fragment ions, known as y and b ions, are generated by the fragmentation of phosphorylated peptides. The retained phosphate group on these ions allows peptide sequence determination where serine, threonine, and tyrosine phosphorylation is observed. This evidence collectively served as a molecular fingerprint to confirm the precise phosphorylation site in peptide sequences. In the typical MS/MS spectrum of the rhodopsin (OPSD/RHO) phosphopeptide ^326^NPLGDEDTSAGK^337^, the y_4_-98 ion and the y_4_ to y_10_ ions encompassed the phosphate group, conclusively pinpointing a phosphorylation site at S334 (Fig. 4C). Similarly, for the OPSV phosphopeptide ^310^ACIMETVCGKPLTDDSDASTSAQR^333^, the y_4_ ion exhibited a high MS intensity in the phosphate group, indicating a phosphorylation site at S328 (Fig. 4D). Finally, for the OPSB phosphopeptide ^333^SPFGDDEDVSGSSQATQVSSVSSSHVAPA^361^, the b_10_-98 ion with the neutral loss of H_3_PO_4_ and the b_9_ ion without phosphate verified a phosphorylation site at S342 (Fig. 4E). Through targeted MS analyses, we accurately mapped the phosphorylation sites within the opsin sequences of RHO, OPSV, and OPSB, further validating the critical roles of opsin phosphorylation during myopia and hyperopia development.

### Structural Insights and Conservation Patterns in Opsin Phosphorylation

**Figure 5.**
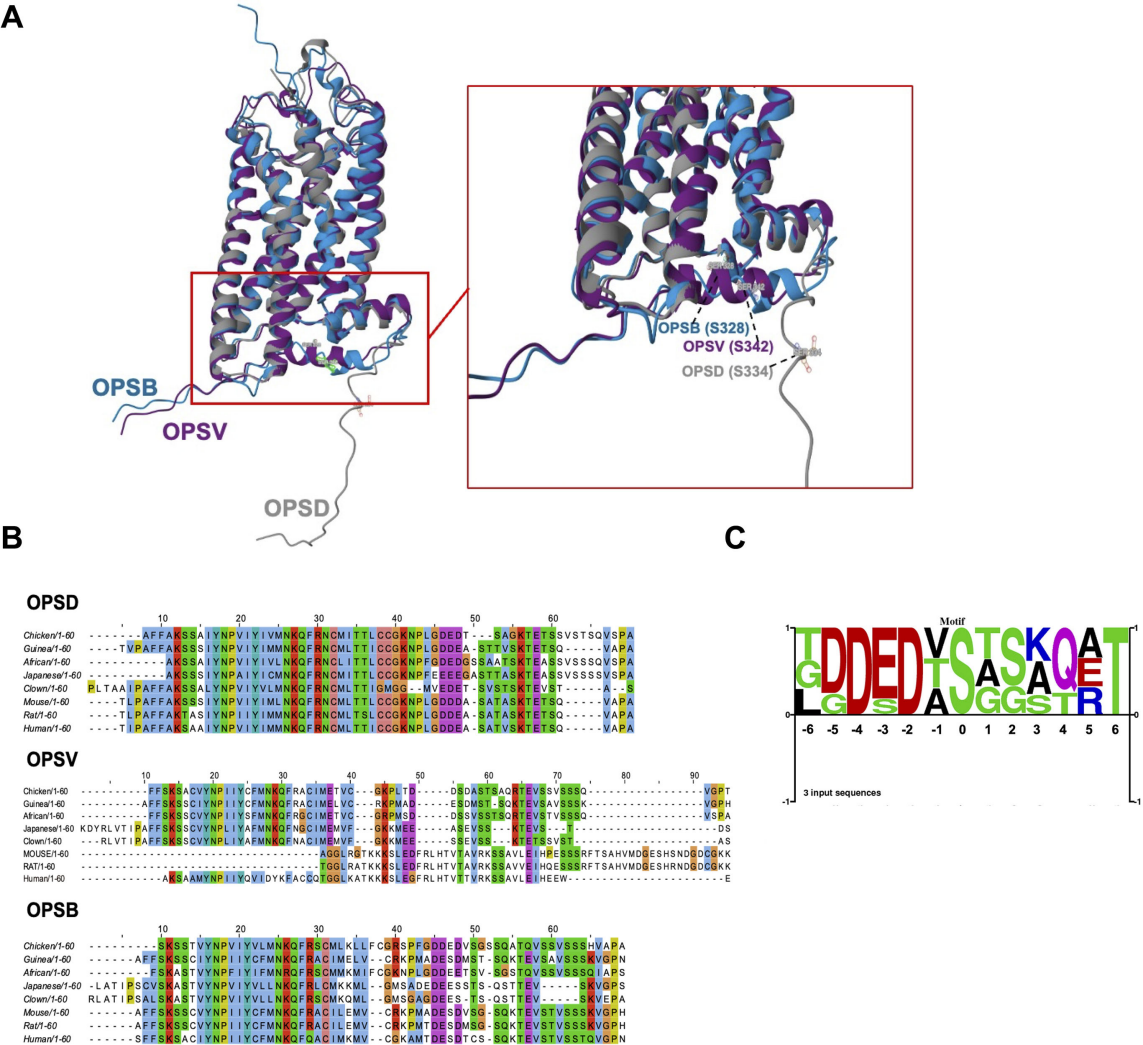
Structural analysis of opsins and the identified phosphorylation site. (**A**) Structures of three opsins based on alpha-fold predictions. (**B**) Conservative analysis of the C-terminal of three opsins. (**C**) Sequence logos of the phosphorylation sites of three opsins.

To illustrate the structural similarities among RHO, OPSB, and OPSV opsins, we performed alpha-fold predictions on the three opsins (Fig. 5A). The predominant domains exhibited a robust per-residue measure of local confidence score (predicted local distance difference test (pLDDT) > 90), indicating high confidence in the structural predictions. Structural alignment indicated high similarities, supported by root-mean-square deviations (RMSDs) ranging from 0.833 to 1.03. It was particularly intriguing that their C-terminal domains with high flexibilities were separated with seven well-defined transmembrane alpha-helices and were suggested to be exposed on surfaces. Notably, these identified phosphorylation sites of S334 in RHO, S328 in OPSV, and S342 in OPSB were all located on the C-terminals. This spatial proximity potentially provides a structural rationale for understanding the phosphorylation mechanisms and their potential disruptive effect on the base of the channel composed of the seven transmembrane alpha-helices. Subsequently, we conducted a sequence alignment to roughly gauge the conservation levels of the C-terminal regions across various lineages (Fig. 5B). The results indicated a high degree of conservation in the C-terminal regions of each opsin across diverse species. Notably, multiple serine residues near the C-terminal in different opsins and species hinted at the functional significance of these amino acid residues in opsins. Through sequence motif analysis (Fig. 5C), we observed a notable prevalence of aspartic acid at positions –4 and –2, alongside a high occurrence of threonine at position +6. This distribution suggests the potential importance of these amino acid residues in the phosphorylation processes of opsins. Because aspartic acid might aid in kinase recognition or binding,^25^ threonine frequently serves as a target for phosphorylation itself.

## Discussion

The retina plays a crucial role in detecting defocus and regulating eye growth independently of the visual cortex, indicating that it possesses intrinsic mechanisms to respond to visual stimuli without relying on higher cortical functions.^26^^,^^27^ This capability is supported by the complex structure of the retina, which is comprised of a diverse array of 136 cell types and over 9200 proteins.^28^^-^^30^ Previous research has demonstrated that dopamine treatment enhances rhodopsin phosphatase activity and increases the rate of rhodopsin dephosphorylation, a mechanism that operates in conjunction with calcium regulation.^31^^–^^33^ We hypothesized that differential phosphorylation of light-sensitive photoreceptors plays a crucial role in developing myopia and hyperopia. Also, retinal opsins are light-sensitive proteins in photoreceptor cells that undergo conformational changes upon photon absorption, initiating the visual signal transduction pathway. To test this hypothesis, we examined the phosphoproteome of chick retinas during the early stages of these refractive errors, specifically after only 3 days of optical treatment.^34^ Our analysis revealed a significant bidirectional phosphorylation modulation in rod and cone photoreceptors. We observed distinct phosphorylation profiles for three photosensitive opsins: rhodopsin, violet-sensitive opsins, and blue-sensitive opsins. In myopic retinas, there was a dephosphorylation of these opsins, suggesting that reduced phosphorylation may be linked to the onset of myopia, potentially correlating with decreased levels of retinal dopamine. Conversely, in hyperopic eyes, we found an increase in the phosphorylation levels of these visual opsins.

Opsins, classified as GPCRs, are crucial in visual transduction.^35^^,^^36^ Phosphorylation of serine residues at the C-terminus of receptors is crucial for regulating receptor desensitization and facilitating the binding of arrestins, which modulate retinal signaling by promoting receptor internalization.^37^^,^^38^ Notably, arrestin-C (*ARR3*), a gene associated explicitly with cone photoreceptors, has been identified as a significant marker for high myopia in humans.^39^^,^^40^ Variations in arrestin expression can influence visual processing and may contribute to refractive errors and eye growth.^41^ We hypothesized that the phosphorylation or dephosphorylation of these residues in opsins may serve vital functions for regulating refractive errors by modulating arrestins and GPCR signaling pathways.^42^ A correlation analysis indicates increased phosphorylation levels in rhodopsin are associated with enhanced arrestin binding.^43^^,^^44^ Our targeted MS experiments confirmed the presence of differential phosphorylated serine residues, specifically, S334 in rhodopsin, S328 in violet-sensitive opsins, and S342 in blue-sensitive opsins. Previous studies have shown that phosphorylation at S334 in rhodopsin occurs following continuous illumination and is linked to the slower phase of rhodopsin dephosphorylation during dark adaptation.^45^ It is a crucial step in the visual cycle necessary for sensitivity recovery. Interestingly, transparent concave and convex spectacle lenses, which induce myopia and hyperopia, respectively, do not alter light illumination intensity, suggesting that the dephosphorylation and phosphorylation of S334 are biochemically mediated rather than influenced by light intensity. During dark adaptation, rhodopsin undergoes dephosphorylation, similar to responses observed under dim light exposure, which has been associated with myopia progression in both animal and human studies.^46^ In addition, the role of dopamine in regulating rhodopsin dephosphorylation suggests that dopamine enhances rhodopsin phosphatase activity.

There is considerable evidence that dopaminergic amacrine cells in the retina play a significant role in releasing dopamine light dependently, with reduced dopamine levels observed in myopic retinas.^47^^–^^49^ In particular, AII (A2) amacrine cells in mammals facilitate the transmission of rod photoreceptor signals from rod bipolar cells to the axonal terminals of cone bipolar cells, highlighting the multifaceted trophic roles of dopamine in modulating retinal circuitry and visual processing.^33^^,^^50^^,^^51^ AII (A2) amacrine cells are well characterized in mammals for their essential role in rod-mediated scotopic vision. In contrast, non-mammalian vertebrates such as chickens (*Gallus gallus*), as demonstrated in this study, do not appear to possess AII (A2) amacrine cells. Instead, amacrine cells in the chick retina represent the most heterogeneous retinal cell class, with single-cell transcriptomic analysis identifying 59 distinct types.^28^ This intrinsic difference reflects the divergent retinal architecture between species, where chick retinas lack the rod-dominant circuitry and instead rely predominantly on cone-mediated photopic vision.^52^ This suggests that non-mammalian vertebrates such as chickens may utilize alternative retinal circuitry mechanisms to fulfill functions analogous to those of mammalian AII (A2) amacrine cells. Furthermore, elevated retinal dopamine is recognized as a “stop” signal that inhibits myopia development, as evidenced by studies involving dopamine agonists and observation in hyperopic eyes.^53^^–^^55^ Although this study did not directly measure retinal dopamine levels, we can infer from existing literature on established LIM and LIH models that our animal model likely exhibits similar dopamine dynamics.^48^ Because dopamine is released by cells in the inner retina, this suggests a novel regulatory mechanism for rhodopsin that originates from the inner retina and affects light receptors.^33^ Although there is limited biochemical evidence regarding the dephosphorylation of S328 in violet-sensitive opsins and S342 in blue-sensitive opsins, emerging research suggests that exposure to short-wavelength lights, particularly violet light (360–400 nm), may suppress myopia progression.^56^^,^^57^ Recent work has demonstrated that violet light suppresses lens-induced myopia (LIM) in mice through violet-sensitive neuropsin (OPN5), which has emission peaks at 380 nm, similar to the violet-sensitive opsin (415 nm) found in chicks.^58^^,^^59^ Similarly, blue-light treatment (451–460 nm) has been found to reduce myopia progression in chicks.^60^^,^^61^ Furthermore, increased time spent outdoors is protective against myopia, possibly due to exposure to sunlight rich in short wavelengths.^62^^,^^63^

The multifactorial etiology of myopia arises from complex interactions between environmental influences and genetic predispositions.^49^ Key factors include developmental pathways involving hypoxia-inducible factor 1 subunit alpha (HIF-1α),^64^ Wnt signaling,^65^ and disrupted lipid metabolism.^66^ Additionally, the interplay between biomechanical forces and genetic factors likely contributes to the phosphorylation changes observed in opsins. Accumulating evidence highlights the role of biomechanical factors, such as tangential forces acting on scleral structure in driving tissue remodeling during myopia progression.^67^ Mechanical stretching of retinal and scleral cells activates multiple molecular signaling pathways, such as transforming growth factor beta (TGF-β),^68^ which mediates cytoskeletal remodeling, extracellular matrix organization, and cell proliferation.^67^ Understanding these interactions may help elucidate the phosphorylation changes in opsins, particularly through force-induced conformational changes in proteins and the activation of mechanosensitive ion channels. Among these, transient receptor potential channels modulate calcium influx in amacrine cells, which can initiate phosphorylation signaling cascades via activation of calcium/calmodulin-dependent protein kinase II (CaMKII).^69^ Future research should aim to elucidate whether the observed phosphorylation changes represent adaptive responses to myopia progression or are causative factors driving the condition, as well as their broader implications for the pathophysiology of refractive errors. Additionally, validating these phosphorylation alterations across multiple species, such as mice and guinea pigs, will be essential to uncover conserved molecular mechanisms and may identify novel therapeutic targets for the effective management of both myopia and hyperopia.

In summary, our investigation into the phosphoproteome of chick retinas during the early stages of refractive errors revealed bidirectional modulation of phosphorylation among rod and cone photoreceptors. Notably, we identified signature patterns of dephosphorylation at S334 in rhodopsin, as well as S328 in violet-sensitive opsins and S342 in blue-sensitive opsins. In contrast, hyperopic eyes exhibited increased phosphorylation levels across these visual opsins. These findings will promote our understanding of the relationship between photosensitive opsins and visual stimuli in the context of manipulated eye growth, shedding light on the molecular mechanisms underlying refractive error development.

## Supplementary Material

Supplement 1
