# Supplementary material for: Bidirectional Phosphorylation Changes in Opsins Associated With Early Myopia and Hyperopia Signal Regulation by Phosphoproteomics
Source: Invest Ophthalmol Vis Sci. 2025 Jul 30;66(9):70. doi: 10.1167/iovs.66.9.70 (PMC12315934; doi:10.1167/iovs.66.9.70)

Supplementary Information

Bidirectional Phosphorylation Changes in Opsins associated with Early Myopia and Hyperopia Signal Regulations by Phosphoproteomics

Yang Yang, Ying Hon Sze, Houjiang Zhou, Winky Wing Man Ko, Yuanliang Zhang, Kecheng Li, Qi Zhang, King Kit Li, Trevor C. Charles, Chi-ho To, Qian Zhao, Thomas Chuen Lam

Table of content

Fig. S1: Body weight, anterior chamber depth, and lens thickness in chicks..1
Fig. S2: Benchmark and optimization of phosphopeptides enrichment...2

Fig. S1: Body weight, anterior chamber depth, and lens thickness in chicks.
a) Bar chart of chick's body weight, showing no significant differences in age-matched comparisons between groups in the TMT-10plex experiment. A significant and consistent disparity in body weight was observed after 3 days of growth (+5.4 g, $p<0.01$). **b**) Anterior chamber depth (ACD) exhibited no statistically significant differences. **c**) No significant differences of lens thickness (LT) at day 7(0) between groups. After 3 days of growth, significant variations were observed with LIM (+75 µm, $p<0.05$), LIH (-82 µm, $p<0.01$), and between groups (LIM-LIH) by day 10 (+116 µm, $p<0.001$). **d**) Body weight for chicks, with no significant differences in age-matched comparison between groups for PRM experiment. A significant, and consistent differences were observed after growing for 3 days in LIM (+15 g, $p<0.001$) and LIH (+19 g, $p<0.001$). **e**) No statistically significant differences were observed in ACD between group at day 7(0) and LIH group after 3 days of treatment. Significant variations were observed in LIM after 3 days (+109 µm, $p<0.001$), and between group (LIM-LIH) by day 10 (73 µm, $p<0.01$). **f**) No statistically significant differences were observed in lens thickness (LT). There was no significant interaction between the effects of age and body weight, or ACD. However, a statistically significant interaction between the lens thickness on age [$F(1, 16) = 18.19$, $p<0.001$]. Statistical analysis was conducted using a two-way ANOVA with Fisher's LSD test. "ns" denotes not significant. * $p<0.05$, ** $p<0.01$, *** $p<0.001$.

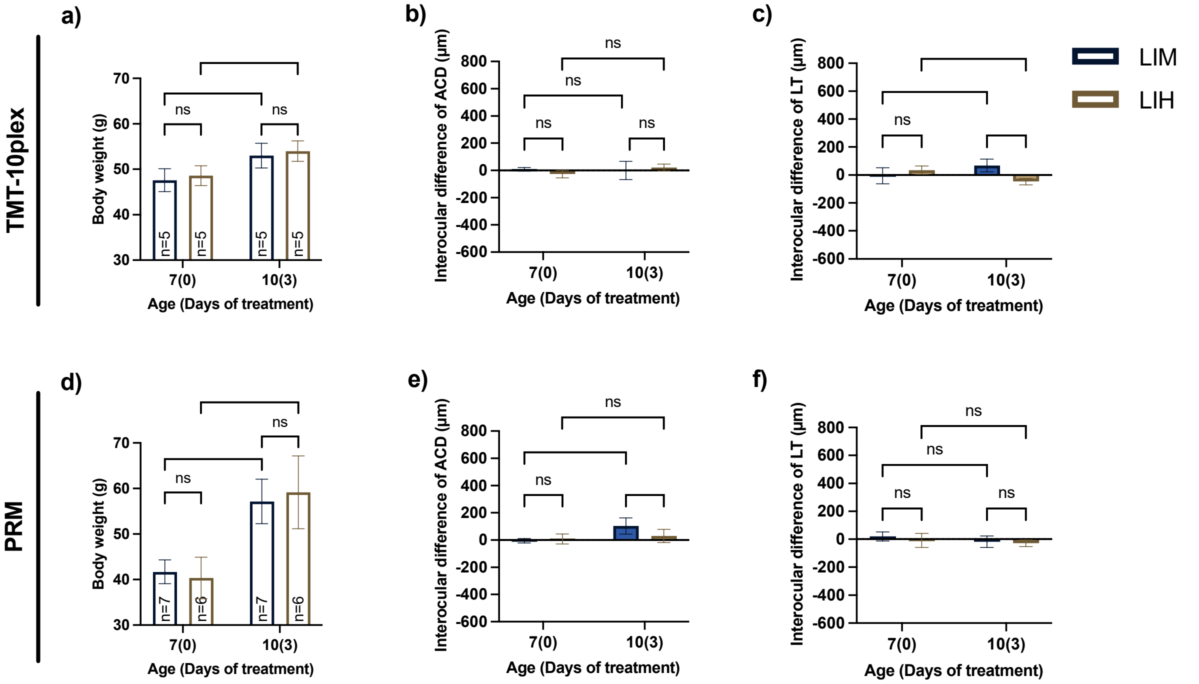

Fig. S2: Benchmark and optimization of phosphopeptides enrichment

To optimize the performance of phosphopeptides enrichment procedure, peptide loading efficiency was evaluated using four titanium dioxide (TiO2) beads-to-peptide ratios of 1:1, 1:2, 1:4, and 1:8 (w/w), with tryptic digested peptides starting from 200 µg protein extracted from chick retinas (n=4). The ratio 1:4 (w/w) was used to prepare the LIM and LIH treated chick retina samples. The bar chart presented the number of identified phosphopeptides (blue bar) and the ratio of phosphopeptides in the mixture (red dot).

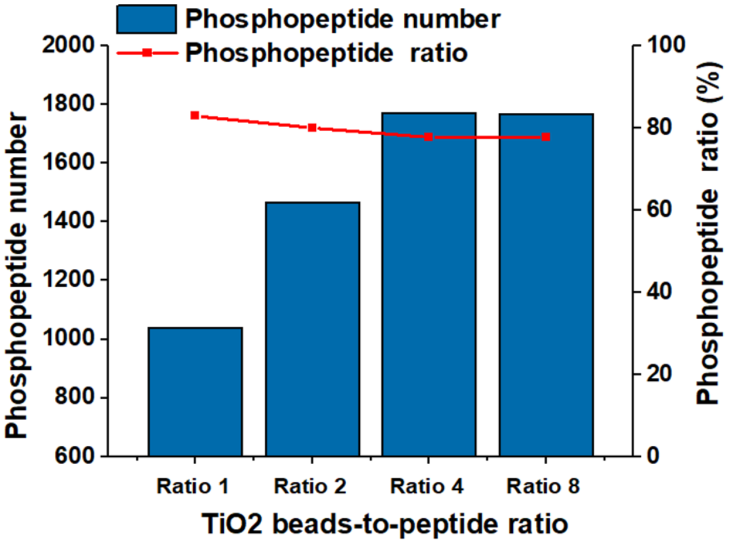

Supplement: Supplement 1 [file iovs-66-9-70_s001.pdf]
